# Reliable *Drosophila* Body Fat Quantification by a Coupled Colorimetric Assay

**DOI:** 10.1371/journal.pone.0023796

**Published:** 2011-09-09

**Authors:** Anja Hildebrandt, Iris Bickmeyer, Ronald P. Kühnlein

**Affiliations:** Forschungsgruppe Molekulare Physiologie, Abteilung Molekulare Entwicklungsbiologie, Max-Planck-Institut für biophysikalische Chemie, Göttingen, Germany; Stockholm University, Sweden

## Abstract

Factors and mechanisms controlling lipometabolism homeostasis share a remarkable evolutionary conservation between humans and *Drosophila* flies. Accordingly, the *Drosophila* model has been successfully used to understand the pathophysiology of human metabolic diseases such as obesity. Body fat stores in species as different as humans and flies consist of neutral lipids, mainly triacylglycerols. Changes in body fat storage are a diagnostic phenotype of lipometabolism imbalances of genetic or environmental origin. Various methods have been developed to quantify *Drosophila* body fat storage. The most widely used method adopts a commercial coupled colorimetric assay designed for human serum triacylglycerol quantification, which is based on glycerol content determination after enzymatic conversion of glycerides into glycerol. The coupled colorimetric assay is compatible with large-scale genetic screen approaches and has been successfully applied to characterize central regulators of *Drosophila* lipometabolism. Recently, the applicability of the coupled colorimetric assay for *Drosophila* storage fat quantification has been questioned in principle. Here we compare the performance of the coupled colorimetric assay on *Drosophila* samples with thin layer chromatography, the “gold standard” in storage lipid analysis. Our data show that the presented variant of the coupled colorimetric assay reliably discriminates between lean and fat flies and allows robust, quick and cost-effective quantification of *Drosophila* body fat stores.

## Introduction

Human lipometabolism disorders such as obesity are severe health hazards and a menacing burden of health care systems [1]. The pandemic spread of overweight and obesity in human populations during the last few decades has provoked increased basic research efforts to explore the genetic and environmental contributions of lipopathologies. Model organisms from yeast to mammals have been employed to unravel the genetic, cellular and physiological basis of lipometabolism (reviewed in [2], [3], [4], [5], [6], [7], [8], [9], [10], [11], [12], [13]).

The fruit fly *Drosophila melanogaster* proved to be a particularly valuable model system, which offers a unique experimental toolbox including genetic screens to identify the genetic basis of body fat storage control. As in mammals, body fat in flies is composed of neutral lipids, mainly triacylglycerols (TAGs), which are stored in intracellular organelles of adipose tissue called lipid droplets. These biochemical and cell biological similarities reflect a remarkable evolutionary conservation of the underlying factors and mechanisms of lipid storage control from flies to man [3], [11].

The body fat content of flies can vary widely and serve as a sensitive diagnostic phenotype indicating imbalances in lipometabolism homeostasis. Various techniques have been used to quantify fat storage in flies. Among them are semi-quantitative techniques such as fat staining by lipophilic dyes in fixed or live *Drosophila* tissues or on histological sections [14], [15]. And there are quantitative methods such as homogenate TAG analysis by thin layer chromatography (TLC; [16], [17]) or mass spectrometry lipid profiling [18], [19], [20]. The most widely used method for storage fat quantification in fly homogenates adopts a commercial coupled colorimetric assays (CCA) developed for human serum TAG analysis [15], [21], [22], [23], [24], [25], [26]. CCA has been successfully applied to characterize central regulators of the *Drosophila* lipometabolism including the Brummer lipase [23] and fly perilipins [22], [27].

However, the applicability of the CCA to reliably quantify storage fat from *Drosophila* homogenates has recently been questioned in principle [28].

Here we directly compare body fat quantification by a *Drosophila* variant of CCA to TAG quantification by TLC using fly homogenates as samples. Our data show that the presented variant of the CCA reliably detects diet- or genotype-dependent storage fat differences between obese and lean flies.

## Results

**Figure 1:**
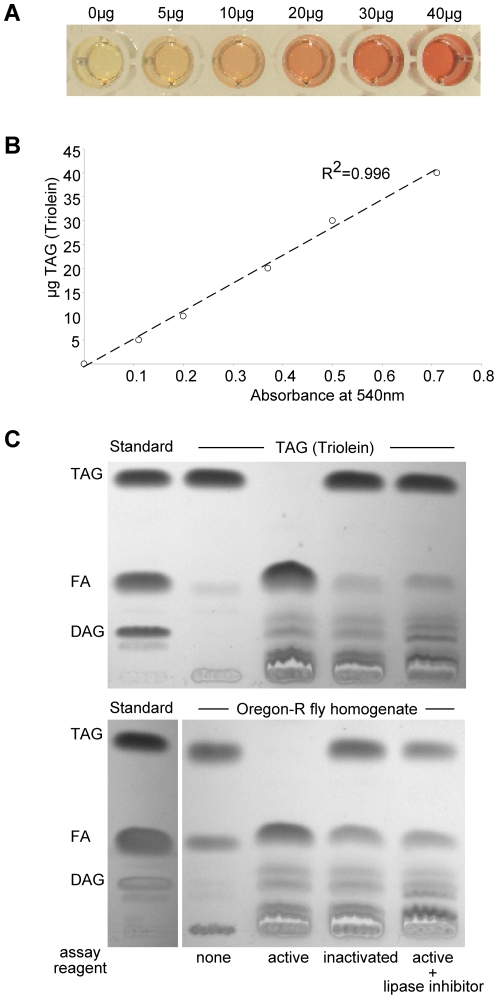
Reliable TAG quantification by the coupled colorimetric assay. Linear absorbance increase of triolein (0–40 µg) measured by CCA is shown by photographic imaging (**A**) and spectrophotometric quantification at 540 nm (**B**). **C** TLC analysis shows complete cleavage of pure TAG (40 µg triolein; upper panel) and storage TAG of *Drosophila* homogenates (lower panel) after CCA with active but not with heat- or lipase inhibitor-inactivated assay reagent.

Commercial CCAs for human serum TAG quantification are based on a chain of enzymatic reactions and essentially measure the glycerol content of the sample. In the first reaction lipoprotein lipase cleaves off the fatty acid (FA) chains from TAGs. Accordingly, the solubility of the hydrophobic TAGs in aqueous fly homogenates and their accessibility by lipoprotein lipase are critical parameters for the complete and accurate quantification of TAGs in this assay. To address the general applicability of TAG quantification using the presented *Drosophila* variant of CCA, 0–40 µg triolein were subjected to the assay and the spectrophotometric absorbance measured at 540 nm. As shown in Figure 1A and 1B, CCA results in a linear increase (R^2^ = 0.996) of absorbance values indicating that this assay allows reliable fat measurement in this concentration range (for photodensitometric quantification of the same amounts of TAG following TLC see Fig. S1). Consistently, TLC analysis of a triolein sample subsequent to CCA development proves the complete degradation of TAG and a corresponding increase of FAs (Fig. 1C, upper panel). As expected for the lipase-dependent cleavage during CCA, TAG deacylation is blocked by heat-inactivation or by adding the lipase inhibitor Orlistat prior to CCA assay development (Fig. 1C). Importantly, also the endogenous storage TAGs of homogenized Oregon-R flies are completely deacylated during CCA (Fig. 1C, lower panel). Taken together these data demonstrate that the presented *Drosophila* variant of CCA reliably quantifies TAGs in a concentration range relevant for body fat measurements in flies.

**Figure 2:**
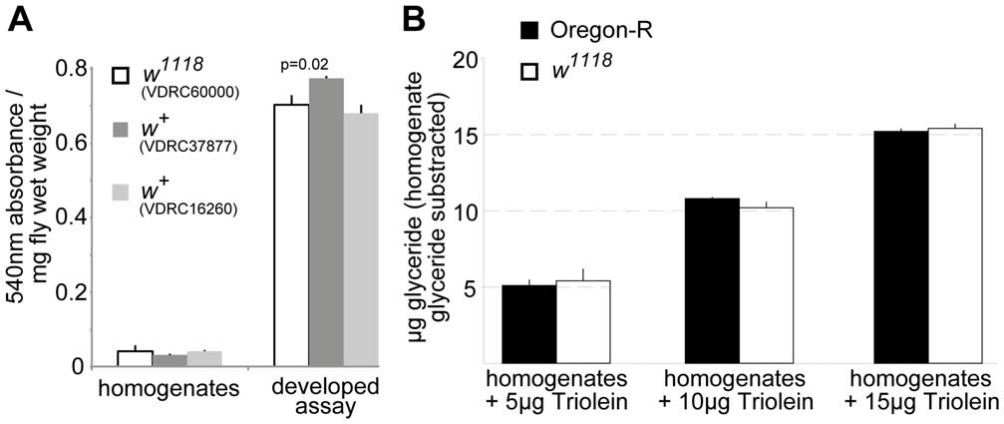
*Drosophila* eye pigments do not adulterate TAG quantification by the coupled colorimetric assay. **A** Absorbance of homogenates from white-eyed *w^1118^* flies compared to genetically matched red-eyed (*w^+^*) transgenic flies prior to and after CCA development. Eye pigments do not contribute significantly to the homogenate absorbance at 540 nm. **B** Eye pigment-independent precise TAG quantification in homogenates from red-eyed Oregon-R and white-eyed *w^1118^* flies supplemented with increasing amounts of triolein. Note: p value is only shown for significant differences compared to *w^1118^*.

End product of the enzymatic reactions in the CCA is a quinoneimine dye, which absorbs in the red part of the visible light spectrum [29]. This fact has raised concerns, that the water-soluble red eye pigments of *Drosophila* could adulterate fat quantifications by CCA, which are based on absorbance measurement at 540nm [28]. However, using our CCA variant there is no statistically significant absorbance difference at 540 nm between white-eyed *w^1118^* flies and genetically matched red-eyed (*w^+^*) transgenic flies (Fig. 2A). Moreover, homogenate absorbance is equivalent to only a minor fraction of the total absorbance at 540 nm after CCA assay development (Fig. 2A). We conclude that differences in eye pigmentation cannot affect the accuracy of the CCA assays. Yet, subtraction of homogenate absorbance (blank subtraction) is an advisable corrective in the presented *Drosophila* CCA protocol.

Eye pigmentation difference has been claimed to possibly influence the sensitivity of CCA [28]. To address this question, fly homogenates supplemented with increasing amounts of exogenous TAG were measured. Figure 2B illustrates that CCA precisely quantifies 5, 10 or 15 µg triolein added to fly homogenates from red-eyed Oregon-R flies or white-eyed *w^1118^* flies, respectively. Collectively, these data demonstrate that the flies' red eye pigments do not interfere with the accurate quantification of TAGs in fly homogenates using the presented *Drosophila* variant of CCA.

**Figure 3:**
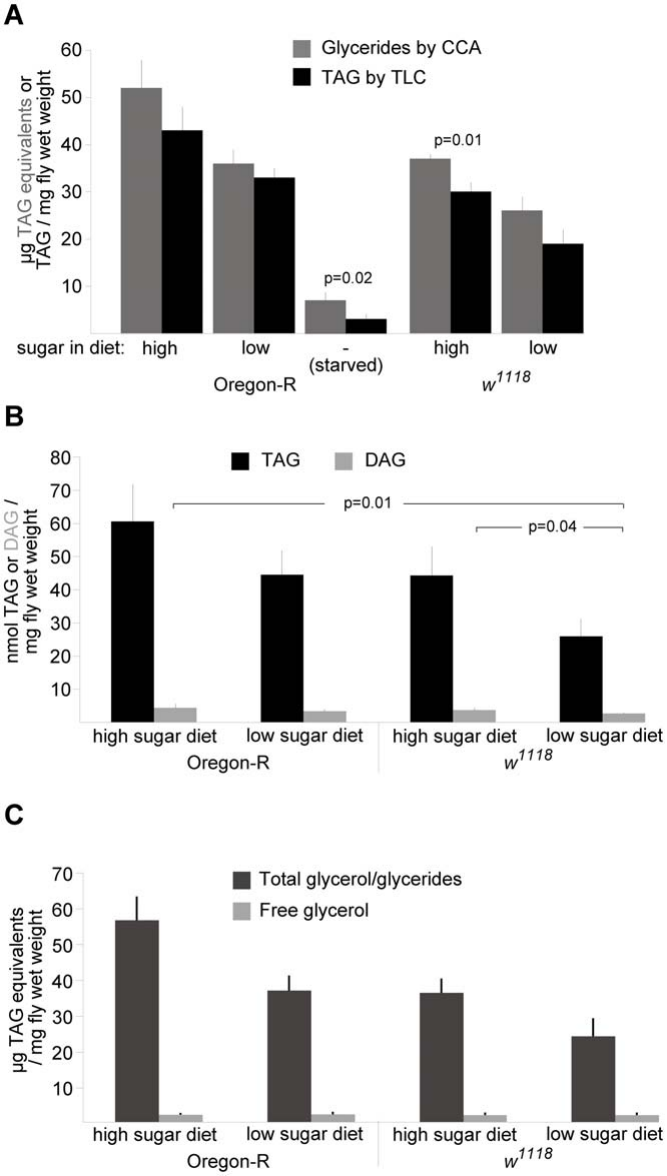
The coupled colorimetric assay reliably predicts storage fat differences between lean and fat flies. **A** Close correlation between total triglyceride equivalent content (determined by CCA) and TAG content (determined by TLC) of flies representing a wide range of genotype- or diet-dependent storage fat. **B** DAG represents a minor fraction of the total TAGs and DAGs in fly homogenates according to TLC quantification. **C** Free glycerol accounts for a small, genotype- and diet-independent fraction of absorbance in the CCA assay. Note: Difference in free glycerol content between genotypes is statistically insignificant.

It is important to note that due to the nature of the enzymatic reactions involved, CCA acts as a general glycerol/glyceride assay with no selectivity for storage fat consisting of TAG. During the CCA reaction development TAGs, DAGs and MAGs are deacylated and, together with free glycerol, add up to a collective glycerol pool, which is eventually quantified. Accordingly, the sample composition determines to what extent the total glycerol/glycerides quantified by this assay matches the storage TAG content. To answer this question for fly homogenates, we directly compared the total glycerol/glyceride content determined by the CCA with the TAG content determined by TLC. *Drosophilae* of different genotypes fed on high or low sugar diet, as well as starved flies were analyzed to cover a broad range of fly storage fat levels. As expected, the measured total glycerol/glyceride content exceeds the TAG values (Fig. 3A). With the exception of the very lean starved flies the TAG content accounts for the vast majority (85% on average) of the total glycerol/glyceride content determined by the CCA. Importantly, there is a close, genotype- and diet-independent correlation between total glycerol/glycerides and TAGs (differences 3–9 µg triglyceride equivalents/mg fly wet weight; TAG content range 3–43 µg/mg fly wet weight). This correlation suggests a fairly constant pool of non-TAG glycerides and free glycerol in flies. To directly determine the relative abundance of the CCA substrates TAG, DAG and free glycerol in homogenates of fat and lean flies they were quantified by two different methods. On one hand TAG and DAG were quantified by TLC, and on the other hand free glycerol and all CCA substrates were quantified by the Sigma Free Glycerol Reagent [30] and by the Sigma Triglyceride Reagent combined with the Free Glycerol Reagent [31], respectively. As shown in Fig. 3B, DAGs account for only 6–9% of the additive TAG and DAG content of Oregon-R or *w^1118^* flies fed on high- or low-sugar diet. Although the absolute differences in DAG content are statistically significant for some selected genotype/diet conditions (Fig. 3B) there is no significant genotype- or diet-dependent difference between the DAG fractions of any of the flies tested. This finding suggests, that storage fat measurements of fly homogenates by CCA can be corrected for the DAG content by a genotype- and diet-independent subtraction factor.

Similarly to DAG, also free glycerol represents only a small, diet- and genotype-independent fraction of all molecular species detected by the CCA in fly homogenates (Fig. 3C). The constant free glycerol content in fly homogenates contributes only 4–8% to the total CCA determined TAG values of fat and lean flies, respectively. Importantly, the absolute free glycerol content of these different flies varies in a narrow range between 1.8 and 2.1 µg triolein equivalents per mg fly wet weight and differences are statistically insignificant in all genotypes and feeding conditions tested. Therefore we conclude that an overestimation of the actual TAG content of flies due to endogenous free glycerol in the homogenate is small in CCA assays and can be largely corrected by subtracting of an average value.

Collectively, these data demonstrate that CCA measurements reliably quantify the storage fat content of fly homogenates. Both, free glycerol and DAGs are only minor contributors to the total CCA signal intensity, which can be adjusted by an average value correction. Similarly, the red eye pigments of flies are a minor CCA interference factor, which can be eliminated for by blank subtraction and does not compromise the sensitivity of the assay. The presented data provide evidence that the applied *Drosophila* variant of CCA robustly discriminates between lean and fat *Drosophilae* and represents a first choice method for the growing field of lipometabolism research in flies.

## Discussion

The recent years witnessed an increasing interest in employing the *Drosophila* model system in lipometabolism research. Variations in fly total body fat content have been scored as diagnostic lead phenotype to unravel the influence of environmental factors such as food composition and particularly the genetic architecture of lipohomeostasis. Noninvasive, indirect methods such as body buoyancy determination in liquid [19] or starvation survival time [23] have been used as predictors of body fat content. Cellular fat storage has been measured more directly by image-based lipid droplet quantification of embryonic *Drosophila* tissue culture cells [32], [33]. Yet, due to poor accessibility of the various fat body depots in the adult fly, total body fat content has been rarely estimated by an image-based method at this ontogenetic stage [34]. Therefore, quantification of the adult *Drosophila* body fat content largely relies on biochemical analysis of fly homogenates. Among the methods in use only CCA is equally suitable for fly fat analysis from the single gene to the large genetic screen format [22], [35].

CCA sample preparation and measurement starting from adult *Drosophila* consists of several steps i.e. fly homogenization/lipid emulsifying, inactivation of endogenous enzymes, cellular debris removal, enzymatic assay development and spectrophotometric quantification. Several parameters of these experimental steps e.g. the detergent concentration and the mode of homogenization during step 1 or the number of flies per assay (Fig. S2) are critical for the reliable quantification. Variation of these critical parameters might explain part of the substantial discrepancies between CCA quantifications of identical fly genotypes reported in the literature (compare e.g. *w^1118^* CCA quantifications in this work to [35]). Notably, differences in fly husbandry including food composition represent an additional, CCA-independent source of variability among body fat quantifications of the very same fly stocks (see e.g. Fig. 3). Finally, the polygenic nature of the traits influencing the fly body fat content call for carefully matched genetic controls to reliably assess the regulatory influence of individual gene activities on body lipid stores.

Given the biochemical characteristics of the CCA, this method is not selective for storage fat. But we provide evidence that the fraction of the CCA signal, which is not attributable to storage fat, is small and predictable in homogenates from genetically and nutritionally heterogeneous flies. It should be emphasized, however, that conditions are conceivable, which substantially alter the relative ratios of the glyceride subclasses in flies and accordingly will influence the accuracy of the storage fat prediction by CCA. Therefore, it is mandatory to confirm CCA quantifications of *Drosophila* body fat content by one or more independent methods such as histological lipid staining, TLC or mass spectrometry-based lipid profiling. Fly studies involving complementary methods corroborate the accuracy of CCA quantifications (for example see [21], [23], [26], [27]). Accordingly CCA is, in due consideration of its limitations, currently the quickest and most cost-effective method of choice for fly lipometabolism studies from single gene analyses to large-scale screens.

## Materials and Methods

### Fly techniques

The Oregon-R (BDSC#2376), *w^1118^* (VDRC#60000; RKF1084) and *w^+^* (VDRC#37877 and VDRC#16260) fly stocks originate from the Bloomington *Drosophila* stock center and the Vienna *Drosophila* RNAi center, respectively. Flies were propagated at 25°C on a high sugar diet [23] and males flies were fed for six days after hatching at 25°C under 12 h∶12 h light/dark conditions on the same food (Oregon-R in Fig. 1C, *w^1118^* in Fig. 3), on low sugar diet (1% sucrose, 1% yeast extract, 1.5% agar, 0.3% proprionic acid, 0.225% Nipagin; Oregon-R in Fig. 3) or subjected to 36 hours water-only starvation after six days feeding on high sugar diet (Fig. 3A). All flies were snap frozen and stored at −20°C before use.

### Lipid analysis

If not described differently the wet weight of cohorts of eight (CCA) or five (TLC) male flies per replicate were determined on a Sartorius MC5 balance before the flies were subjected to lipid analysis.

### Coupled colorimetric assay (CCA)

CCA quantification of *Drosophila* homogenates was essentially done as described in [22]. If not described differently eight flies per replicate were homogenized in a 2 ml screwcap tube containing 1 ml 0.05% Tween-20 and a ceramic cylinder using a peqlab Precellys 24 instrument (10 sec at 5000 rpm). Homogenates were heat-inactivated (5 min at 70°C) and debris pelleted in a Beckmann GS6KR centrifuge (3 min at 3500 rpm). Of the supernatants 50 µl samples were transferred to a 96 well microtiter plate and homogenate (blank) absorbance was measured at 540 nm in a Biorad Benchmark Microplate Reader. Prewarmed Triglyceride solution (200 µl; Thermo Fisher Scientific #981786) was added to each homogenate sample and incubated at 37°C with mild shaking for 30–35 min. Total absorbance at 540 nm was measured and corrected by subtraction of blank and substrate absorbance prior to triglyceride equivalent content calculation using 0–40 µg of triolein (Sigma T7140) as TAG standard, which was treated like the samples.

For experiments with inactive CCA reagent shown in Fig. 1C the Triglyceride solution was heat-inactivated (5 min at 96°C) or incubated with 200 µM of the lipase inhibitor Orlistat (Sigma O4139) prior to use.

For homogenate absorbance determination prior to CCA assay (Fig. 2A), the 540 nm absorbance of 250 µl 0.05% Tween-20 was subtracted as blank value. Homogenate absorbance values were calculated per mg fly wet weight.

For experiments shown in Fig. 2B, 16 flies per replicate were homogenized in 1 ml 0,05% Tween-20. Homogenate supernatants (150 µl) were added to equal volumes of 0.05% Tween-20 containing increasing amounts of triolein and treated once more in the peqlab Precellys 24 instrument as described. Aliquots (50 µl) of the resulting homogenate samples were subjected to CCA measurement as described.

Shown are representative experiments with average values of triplicate measurements and corresponding standard deviations. Experiments were repeated at least twice.

For fly free glycerol content determination eight male flies were homogenized in 0.5 ml 0.05% Tween-20 as described above. Free glycerol content of 50 µl homogenate supernatants was determined with the Free Glycerol Reagent (Sigma F6428) using 0–50 µg triolein equivalents (Glycerol Standard Solution, Sigma G7793) as standard. Total free glycerol and glyceride content was determined by diluting 25 µl of the aforementioned homogenate with 25 µl 0.05% Tween-20 before using the Free Glycerol Reagent combined with the Triglyceride Reagent (Sigma T2449+F6428) using 0–40 µg triolein as standard. Free glycerol content and total free glycerol+glyceride content both expressed as µg triolein equivalent/mg fly wet weight were calculated as described above.

Shown are average values of triplicate measurements of three independent experiments.

### Thin layer chromatography (TLC)

Fly lipids were extracted according to Bligh and Dyer [36]. Five flies per replicate were homogenized in 150 µl methanol, 75 µl chloroform and 60 µl water in a Bioruptor sonifier (15 min with alternating 45 sec on/off intervals, low intensity setting; www.diagenode.com) or in the peqlab Precellys 24 instrument (10 sec 5000 rpm) using 1.4 mm ceramic beads (peqlab 91-PCS-CK14S). Lipids were extracted from the homogenates for 1 hour at 37°C before 75 µl chloroform and 75 µl 1 M KCl were added. Phase separation was achieved by centrifugation (Eppendorf 5417C; 2 min 3000 rpm) and the chloroform phase solvent was evaporated in a SpeedVac concentrator (Thermo Savant ISS110). Lipid pellets were resuspended in 60–70 µl chloroform/methanol (1∶1). For fat extraction after CCA the samples were extracted with 500 µl methanol and 250 µl chloroform for 15 min at 37°C before adding 250 µl chloroform and 250 µl 1 M KCl and lipid recovery as described above. Lipid extracts from CCA samples were separated by TLC as described below using 20 µg each of triolein, pentadecanoin and stearic acid as lipid standards.

Lipids extracted from 1 mg fly wet weight were separated on high performance thin layer chromatography (HPTLC) plates (Merck 105633) using n-hexane/diethylether/acetic acid (70∶30∶1, v/v/v; Merck) as liquid phase along with the following standard lipids: triolein (TAG; Sigma T7140), pentadecanoin (DAG; Sigma D8508), stearic acid (FA; Fluka 85679). Plates were air dried, dipped into 8% (w/v) H_3_PO_4_ containing 10% (w/v) copper (II) sulfate pentahydrate and charred for 10 min at 180°C on a hot plate (Gerhard H22 electronic). Fly lipid classes were quantified by photodensitometry (FujiFilm LAS-1000 and Image Gauge V3.45) scaled to a dilution series of the corresponding lipid standard (5–80 µg triolein; 1–16 µg pentadecanoin).

Depicted in Fig. 3A are representative experiments with average values of triplicate measurements and corresponding standard deviations. Experiments were repeated at least twice. Shown in Fig. 3B are average values of triplicate measurements of two independent experiments.

To determine the glyceride composition of fly homogenates the TAG and DAG content of flies was determined by TLC and the free glycerol content by CCA. Relative abundance of the glyceride classes was calculated using the following (average) molecular weights: glycerol (92,1 g/mol), triglycerides (844,96 g/mol), diglycerides (562,5 g/mol) and expressed as nmol/ mg fly wet weight.

### Statistical analysis

If not stated otherwise mathematical significance of differences between datasets was analyzed using the unpaired t test and expressed as p values. Curve fitting of the standard curves was done in Microsoft Excel by adding trendlines (regression type “linear” for CCA and regression type “power” for TLC) to the standard data points.

## Supporting Information

Figure S1 **Photodensitometric quantification of TAG by TLC.** Photographic image (**A**) and photodensitometric quantification (**B**) of TLC-separated and charred TAG (0–40 µg triolein). Note: Horizontal bars in **B** represent the standard deviations of replicate measurements.
(TIF)

Figure S2 **CCA measurement accuracy depends on the fly number per assay.** Shown are total TAG measurements of six replicates each of cohorts from two to 16 *w^1118^* flies. TAG increase is linear over a wide range of flies per assay. Note the underestimation of TAG values with two flies per assay and the substantial scattering of the values with large cohort sizes. The line represents the expected TAG values based on eight flies per assay measurements proposed in the presented CCA protocol variant.
(TIF)
