# Supplementary figures and images for: Reliable Drosophila Body Fat Quantification by a Coupled Colorimetric Assay
Source: PLoS One. 2011 Sep 9;6(9):e23796. doi: 10.1371/journal.pone.0023796 (PMC3170289; doi:10.1371/journal.pone.0023796)

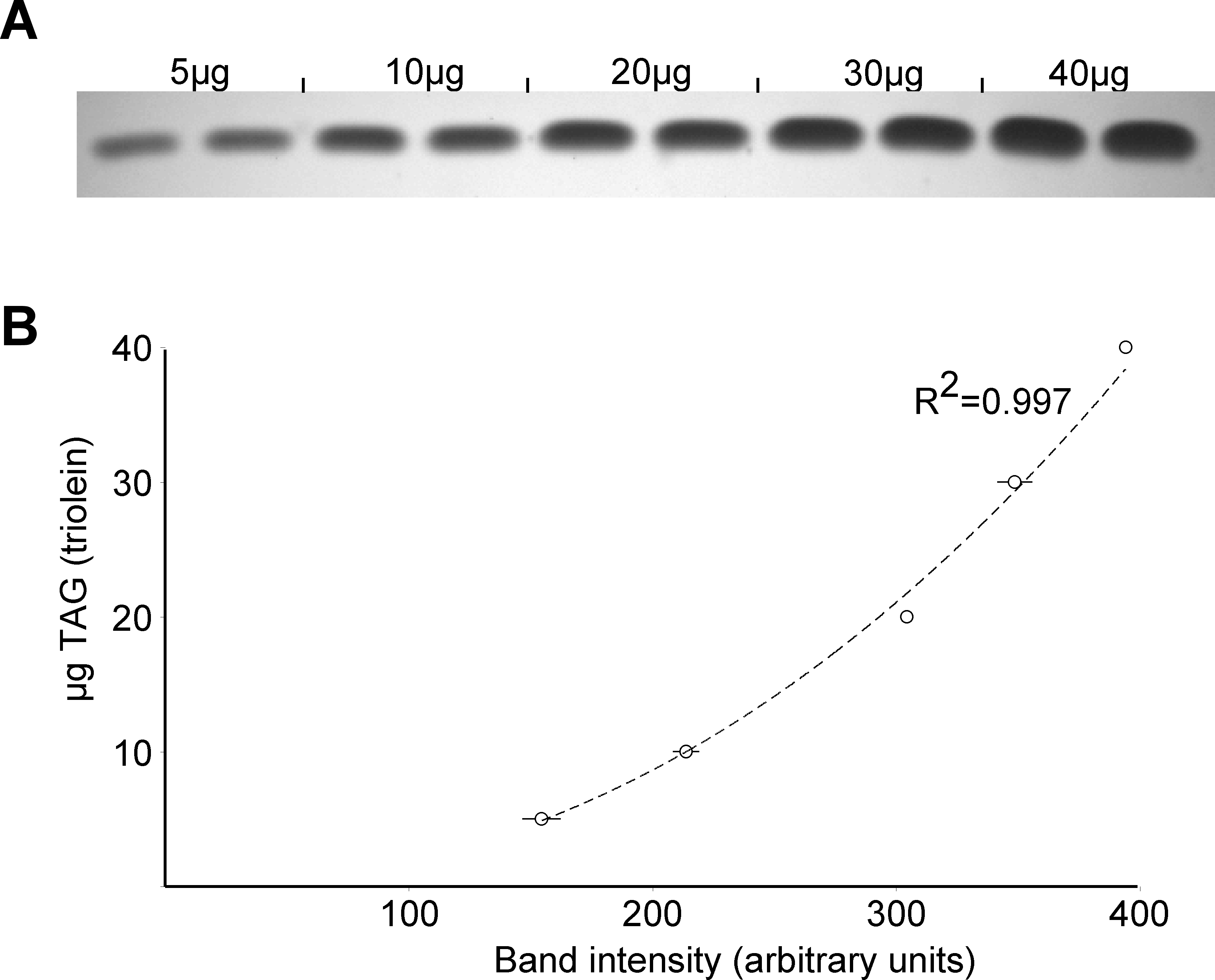

Supplement: Figure S1 — Photodensitometric quantification of TAG by TLC. Photographic image (A) and photodensitometric quantification (B) of TLC-separated and charred TAG (0–40 µg triolein). Note: Horizontal bars in B represent the standard deviations of replicate measurements. (TIF) [file pone.0023796.s001.tif]

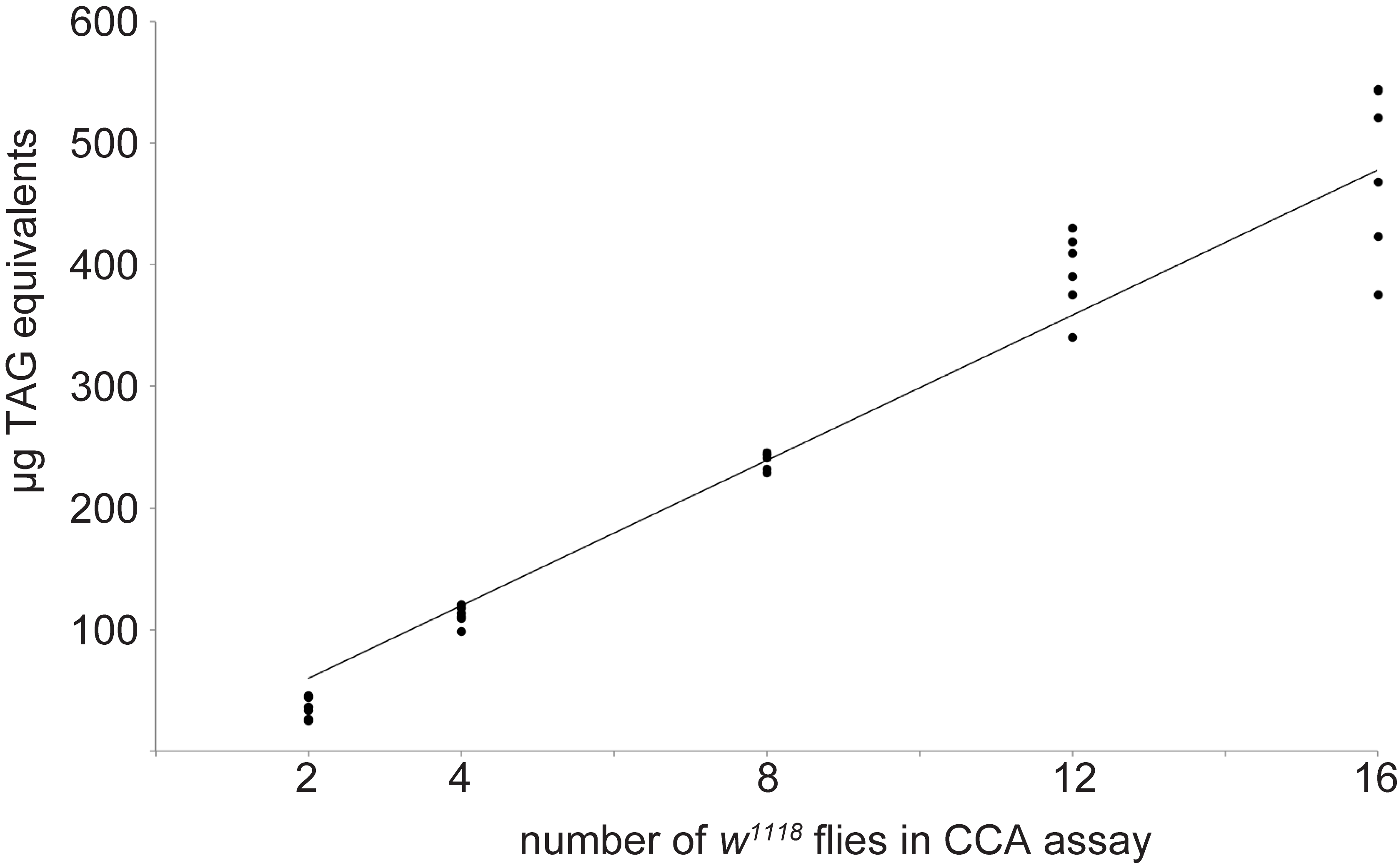

Supplement: Figure S2 — CCA measurement accuracy depends on the fly number per assay. Shown are total TAG measurements of six replicates each of cohorts from two to 16 w1118 flies. TAG increase is linear over a wide range of flies per assay. Note the underestimation of TAG values with two flies per assay and the substantial scattering of the values with large cohort sizes. The line represents the expected TAG values based on eight flies per assay measurements proposed in the presented CCA protocol variant. (TIF) [file pone.0023796.s002.tif]
